# Identification of STOP1-Like Proteins Associated With Aluminum Tolerance in Sweet Sorghum (*Sorghum bicolor* L.)

**DOI:** 10.3389/fpls.2018.00258

**Published:** 2018-02-28

**Authors:** Sheng Huang, Jie Gao, Jiangfeng You, Yanan Liang, Kexing Guan, Siqi Yan, Meiqi Zhan, Zhenming Yang

**Affiliations:** Jilin Province Engineering Laboratory of Plant Genetic Improvement, College of Plant Science, Jilin University, Changchun, China

**Keywords:** aluminum toxicity, STOP1, transcriptional regulation, Al tolerance genes, sweet sorghum

## Abstract

Aluminum (Al) toxicity in acidic soils affects crop production worldwide. C_2_H_2_-type zinc finger transcription factor STOP1/ART1-mediated expression of Al tolerance genes has been shown to be important for Al resistance in *Arabidopsis*, rice and other crop plants. Here, we identified and characterized four STOP1-like proteins (SbSTOP1a, SbSTOP1b, SbSTOP1c, and SbSTOP1d) in sweet sorghum, a variant of grain sorghum (*Sorghum bicolor* L.). Al induced the transcription of the four *SbSTOP1* genes in both time- and Al concentration-dependent manners. All SbSTOP1 proteins localized to the cell nucleus, and they showed transcriptional activity in a yeast expression system. In the HEK 293 coexpression system, SbSTOP1d showed transcriptional regulation of *SbSTAR2* and *SbMATE*, indicating the possible existence of another SbSTOP1 and SbSTAR2-dependent Al tolerance mechanism in sorghum apart from the reported SbMATE-mediated Al exclusion mechanism. A transgenic complementation assay showed that *SbSTOP1d* significantly rescued the Al-sensitivity characteristic of the *Atstop1* mutant. Additionally, yeast two-hybrid and bimolecular fluorescence complementation (BiFC) assays showed that SbSTOP1d interacted with SbSTOP1b and SbSTOP1d itself, suggesting that SbSTOP1 may function as a homodimer and/or heterodimer. These results indicate that STOP1 plays an important role in Al tolerance in sweet sorghum and extend our understanding of the complex regulatory mechanisms of STOP1-like proteins in response to Al toxicity.

## Introduction

Acid soils are widespread and limit crop production all over the world. Aluminum (Al) toxicity is a primary limiting factor in acid soils. At pH below 5, Al (the most abundant form, Al^3+^) inhibits root elongation within a few minutes, which leads to subsequent water and nutrient deficiency (Kochian et al., 2004; Ma, 2007; Bojórquez-Quintal et al., 2017).

To cope with Al stress, plants develop a series of strategies that have been categorized into two main types of Al resistance mechanisms. Al exclusion mechanisms, which are external strategies, aim at preventing toxic Al from entering root cells by exuding organic compounds (e.g., organic acids or phenolics) into the rhizosphere to chelate Al. Al tolerance mechanisms, which are internal strategies, sequester and detoxify Al that enters the plant (Ma, 2000; Ryan et al., 2001; Kochian et al., 2015). The mechanisms of Al-meditated root exudation of organic acids (citrate, malate or oxalate) are well characterized, with involvement of transporters from the Al-activated malate transporter (ALMT) family and the multidrug and toxic compound extrusion (MATE) family (Sasaki et al., 2004; Furukawa et al., 2007; Magalhaes et al., 2007). *SbMATE* in sorghum (and *MATE* in barley, *HvAACT1*) was the first Al resistance gene to be identified. It encodes a citrate transporter that is primarily responsible for Al resistance in sorghum via citrate release (Furukawa et al., 2007; Magalhaes et al., 2007). Subsequently, homologs of MATE were isolated in other species, including AtMATE1 in *Arabidopsis thaliana*, VuMATE1 in *Vigna umbellata* and OsFRD1 in *Oryza sativa* (Liu et al., 2009; Yang et al., 2011; Yokosho et al., 2011). Once Al traverses the external organic compound barrier, it reaches the root cells. In response, plants develop Al tolerance mechanisms that involve other membrane transporters, including Nramps, ABC transporters and aquaporins (Huang et al., 2009, 2010; Negishi et al., 2012, 2013; Li et al., 2014). The cell wall constitutes the first barrier against Al in cells, while some ABC transporters (such as OsSTAR1/OsSTAR2 protein complex) are thought to mediate the efflux of UDP-glucose into the cell wall, which presumably alters the cell wall composition, limiting Al accumulation and reducing Al toxicity (Huang et al., 2009).

Al induces the coordinated expression of multiple Al tolerance genes in plants. Sensitive to proton rhizotoxicity 1 (STOP1) in *Arabidopsis* was isolated and further shown to be a key transcription factor that regulates the expression of a range of Al tolerance genes (including *AtALMT1, AtMATE*, and *AtALS3*) and some proton tolerance genes (Liu et al., 2009; Sawaki et al., 2009). Al resistance transcription factor 1 (ART1) was also identified in rice. In contrast to AtSTOP1, the rice homolog regulates only Al tolerance genes (such as *OsNrat1, OsSTAR1*, and *OsSTAR2*) but not proton tolerance genes (Yamaji et al., 2009). Homologous *STOP1*-like genes have also been characterized in other plant species. These genes all encode a Cys_2_His_2_ (C_2_H_2_) zinc finger protein, but their expression patterns vary. *AtSTOP1* in *A. thaliana, OsART1* in *O. sativa*, and *NtSTOP1* in *Nicotiana tabacum* are constitutively expressed in roots, whereas *VuSTOP1* in *V. umbellata* is upregulated by Al toxicity in a dosage-dependent manner (Yamaji et al., 2009; Ohyama et al., 2013; Fan et al., 2015). AtSTOP2, a homolog of AtSTOP1, was identified in *Arabidopsis* recently. AtSTOP2 activates the expression of some genes for Al- and low pH-tolerance that are regulated by AtSTOP1 (Kobayashi et al., 2014). The distinct roles and/or consociation of AtSTOP1 and AtSTOP2 in Al signaling and regulatory pathways, however, have not yet been clarified.

In this study, four *STOP1*-like genes (*SbSTOP1a, SbSTOP1b, SbSTOP1c*, and *SbSTOP1d*) with diverse expression profiles were identified in sweet sorghum, a variant of grain sorghum (*Sorghum bicolor* L.). SbSTOP1d, which shares the highest identity with AtSTOP1 and OsART1, regulated the transcription of *SbSTAR2*, suggesting the existence of a SbSTOP1-mediated Al tolerance mechanism aside from the previously reported SbMATE-dependent Al exclusion mechanism in sorghum. SbSTOP1d interacted with itself and SbSTOP1b in plants, implying that SbSTOP1d might form a homo- and/or heterodimer to function. Taken together, we characterized homologous SbSTOP1s in sweet sorghum and examined the association between diverse SbSTOP1s, which may help to further clarify the complex signal transduction pathways of STOP1-like proteins in response to Al toxicity.

## Materials and Methods

### Plant Materials, Culture Conditions, and Al Treatments

The sweet sorghum (*S. bicolor* L.) cultivar POTCHETSTRM was used in this study (Zhang et al., 2015). Seeds were surface sterilized with 1% (v/v) NaClO for 20 min, rinsed with deionized water five times, spread on wet filter paper in a Petri dish and germinated for 2 days in darkness at 28°C. The germinated seeds were transplanted into 0.5 mM CaCl_2_ solution at pH 4.5 or 5.8 depending on the treatment. The seedlings were grown in an environmentally controlled growth chamber with a 14 h light (400 μmol m^-2^ s^-1^)/10 h dark photoperiod, 26°C day/22°C night temperatures and 80% relative humidity.

For gene expression pattern analysis, seedlings cultured for 3 days in 0.5 mM CaCl_2_ solution (pH 4.5) were then exposed to a different treatment. For the time-course assay, seedlings were exposed to 0.5 mM CaCl_2_ solution with 15 μM AlCl_3_ for 0, 3, 6, 9, or 24 h (pH 4.5), then the root apices (0–1 cm) were excised. For the Al concentration-dependent assay, seedlings were exposed to 0.5 mM CaCl_2_ solution with 0, 5, 10, 15, or 30 μM AlCl_3_ for 24 h (pH 4.5), then the root apices (0–1 cm) were cut. For the tissue expression pattern assay, seedlings were exposed to 0.5 mM CaCl_2_ solution with 0 or 15 μM AlCl_3_ for 24 h (pH 4.5) with roots (0–1 cm, 1–2 cm, or 2–3 cm) and shoots excised. For the different metal treatments, seedlings were exposed to 15 μM AlCl_3_, 10 μM CdCl_2_, 0.5 μM CuCl_2_ or 10 μM LaCl_3_ for 24 h (pH 4.5), then the root apices (0–1 cm) were excised. For the low pH treatments, seedlings were cultured in 0.5 mM CaCl_2_ solution (pH 5.8) for 3 days, then exposed to the same solution at pH 5.8, 5.0, 4.5, 4.0, or 3.5 for 24 h. Then, the root apices (0–1 cm) were excised for RNA isolation. Each treatment was analyzed using three biological replicates.

### Sequence Analysis

All sequences were analyzed using BLAST in the sorghum genome database^1^ and NCBI. Sequence alignment was performed using Vector NTI and modified in GeneDoc. The phylogenetic tree was constructed according to the neighbor-joining method using MEGA 5.1.

### RNA Isolation and Quantitative Real-Time PCR

Total RNA isolation, cDNA preparation and quantitative real-time PCR (qRT-PCR) were performed as previously described (Zhang et al., 2015). The gene-specific primers were designed using Primer 5.0 software (Supplementary Table 1). The house-keeping gene β*-actin* (GenBank ID: X79378) was used as an internal control (Zhang et al., 2015). The qRT-PCR was performed using SYBR Premix ExTaq (Takara) in an Mx3005P qPCR system (Stratagene, United States). Thermocycling proceeded as follows: 1 cycle of 30 s at 95°C, 30 cycles of 5 s at 95°C and 20 s at 60°C, and 1 cycle of 60 s at 95°C, 30 s at 55°C, and 30 s at 95°C for the melting curve analysis. The relative expression level of the genes was calculated using the 2^-ΔΔC_T_^ method (Livak and Schmittgen, 2001). The experiment was conducted using three biological replicates.

### Subcellular Localization of SbSTOPs

*Arabidopsis* protoplasts were isolated from 4-week-old plants. Leaves were cut into strips and transferred quickly into the enzyme solution [1% (w/v) cellulase R10, 0.25% (w/v) macerozyme R10, 0.4 M D-mannitol, 20 mM KCl, 20 mM MES pH 5.7 and 10 mM CaCl_2_] for 1 h digestion at room temperature in darkness. Protoplasts were filtered through a 100-micron nylon mesh and centrifuged for 2 min at 100 *g*, rinsed with ice-cold W5 buffer [154 mM NaCl, 125 mM CaCl_2_, 5 mM KCl and 2 mM MES, pH 5.7], and suspended in MMg buffer [0.4 M mannitol, 15 mM MgCl_2_, 4 mM MES, pH 5.7]. Afterward, the protoplasts were ready for transformation. The PEG-mediated protoplast transformation method was used in this study. 10 μl of *35S::YFP-SbSTOP1a* (or *35S::YFP-SbSTOP1b, 35S::YFP-SbSTOP1c* and *35S::YFP-SbSTOP1d*) was mixed with 100 μl protoplasts and 110 μl PEG solution [40% (w/v) PEG4000, 0.2 M mannitol, 100 mM CaCl_2_]. The protoplast/DNA mixture was incubated at room temperature in darkness for 15 min, washed twice with W5 buffer, and incubated in darkness at room temperature for 12–16 h. The fluorescence images were captured using a fluorescence microscope (Axio Observer A1, Zeiss).

### Transcriptional Activity Detection and Yeast Two-Hybrid Assay

To detect the transcriptional activity of SbSTOP1s, the bait vector pBridge expressing SbSTOP1a, SbSTOP1b, SbSTOP1c, SbSTOP1d, SbSTOP1d-NT (1–275 aa) or SbSTOP1d-CT (276–519 aa) fused to the GAL4 DNA-binding domain (BD) was used to transform the yeast strain Y2HGold. Colonies were selected on SD/-Trp-His medium (with or without 3-AT) and cultured for 3 days at 30°C. For the yeast two-hybrid assay, the prey vector pGADT7 expressing SbSTOP1b or SbSTOP1d fused to the GAL4 activation domain (AD) and the bait vector pBridge expressing SbSTOP1d-NT (1–275 aa) fused to the BD were used to co-transform the yeast strain Y2HGold (or the Y190 yeast strain for the β-galactosidase assay). Colonies were selected on SD/-Trp-Leu-His medium and cultured for 3 days at 30°C. The β-galactosidase assay was performed using chlorophenol red-β-D-galactopyranoside (CPRG) as substrate, and Miller units were calculated according to the Yeast Protocols Handbook (Clontech, PT3024-1). The experiment was conducted using three biological replicates.

### HEK293 Coexpression System and Dual-Luciferase Reporter Assay

To examine the transcriptional regulation of *SbMATE* or *SbSTAR2* by SbSTOP1d, the reporter plasmid (*pSbMATE::LUC*-*SV40::REN* or *pSbSTAR2::LUC*-*SV40::REN*) and effector plasmid (*CMV::SbSTOP1d-Myc*) were co-transfected into HEK293 (human embryonic kidney) cells.

HEK293 cells were cultured as previously described (Gao et al., 2015). Cells were maintained in Dulbecco’s modified Eagle’s medium (DMEM) with FBS (10%) and penicillin/streptomycin (1%) in a cell culture flask, T75 (Eppendorf), which was incubated in a 37°C incubator with a humidified atmosphere of 5% CO_2_ in air. When the cell count reached 2 × 10^7^, cells were subcultured in a 6-well plate the night before and grown to 60–70% confluence by the day of transfection. HEK293 cells were transfected with the constructed plasmids (reporter and effector) using the calcium phosphate transfection method as reported (Gao et al., 2015). After 30–48 h, the transfected cells were ready for the dual-luciferase reporter assay.

The dual-luciferase reporter assay was conducted according to the technical manual of the Dual-luciferase Reporter Assay System (Promega, E1910). After removing the growth medium, the transfected cells were gently rinsed with 1 × PBS (pH 7.2, Thermo, 20012050) and lysed in 1 × Passive Lysis Buffer (PLB). The PLB lysate was plated in a 96-well plate with volume ≤20 μl/well. The firefly luciferase activity was measured by adding 100 μl of Luciferase Assay Reagent II (LAR II) to generate a luminescent signal that was measured with a luminometer (Berthold LB960). This reaction was then quenched, and the *Renilla* luciferase reaction is simultaneously initiated by adding 100 μl of Stop & Glo^®^ Reagent to the same well. The Stop & Glo^®^ Reagent also produced a luminescent signal from the *Renilla* luciferase, which served as an internal control. The experiment was conducted using three biological replicates.

### Bimolecular Fluorescence Complementation Assay

Different pairs of plasmids encoding nYFP-SbSTOP1d and cCFP-SbSTOP1b, or encoding nYFP-SbSTOP1d and cCFP-SbSTOP1d were co-transformed into *Arabidopsis* protoplasts. The protoplast preparation and transformation method are described above. The reconstituted YFP fluorescence images were examined by a fluorescence microscope (Axio Observer A1, Zeiss), and the percentage of cells that exhibited bimolecular fluorescence complementation (BiFC) fluorescence signals were calculated. The experiment was conducted using three biological replicates.

### Overexpression of *SbSTOP1d* in the *Atstop1* Mutant

The open reading frame (ORF) of *SbSTOP1d* was amplified and cloned into the pEGAD vector (*35S::LUC-SbSTOP1d*) using the In-Fusion enzyme. The construct was transformed into *Agrobacterium tumefaciens* strain AGL0, which was further introduced into the *Atstop1* mutant using the floral dip method (Clough and Bent, 1998). The transgenic seedlings were first screened with the Basta herbicide, then confirmed by a three-primer PCR-based genotyping using the following primers: LP, 5′- TTCATTGGTGAGAACGACTCC -3′, RP, 5′- ATCTTCTTGTTGGTCGTGGTG -3′, LB, 5′- ATTTTGCCGATTTCGGAAC -3′. An immunoblot assay was performed to examine the expression of the fusion protein LUC-SbSTOP1d. After seeds were surface sterilized and germinated on solid MS medium vertically for 5 days, uniform seedlings were transferred to solid medium containing 4.3 mM CaCl_2_ and 3% sucrose at pH 4.5, with or without 50 μM AlCl_3_ for 2 days, and their root growth was measured. At least 20 seedlings were measured for each treatment and independent experiments were performed three times.

## Results

### Sequence Analysis of SbSTOP1s

**FIGURE 1:**
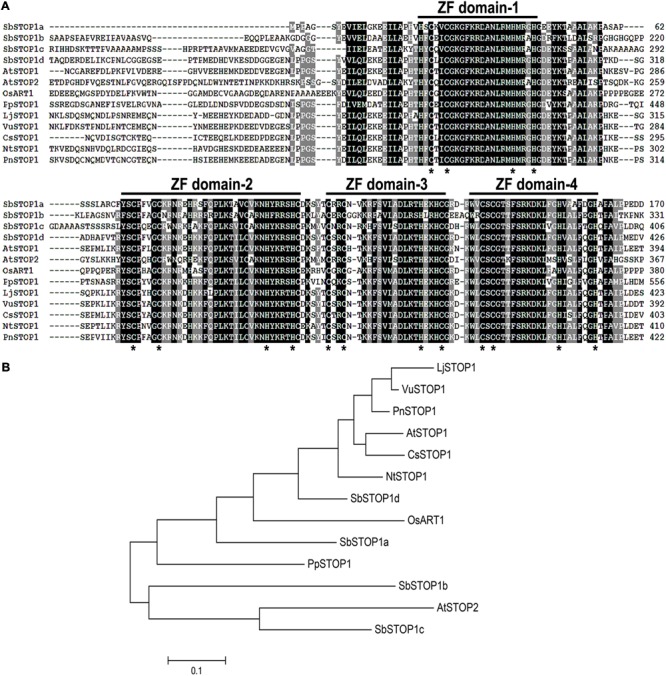
Identification of SbSTOP1s. **(A)** Sequence alignment of the zinc finger domains of SbSTOP1s and homologous proteins from other species, including *Arabidopsis thaliana* (AtSTOP1, At1g34370, and AtSTOP2, At5g22890), *Physcomitrella patens* (PpSTOP1, AB811779), *Nicotiana tabacum* (NtSTOP1, AB811781), *Oryza sativa* (OsART1, AB379846), *Camellia sinensis* (CsSTOP1, AB811780), *Populus nigra* (PnSTOP1, AB811778), *Lotus japonicus* (LjSTOP1, AB811782) and *Vigna umbellata* (VuSTOP1, KP637172). Horizontal lines indicate zinc finger (ZF) domains, and asterisks show conserved Cys_2_His_2_ or Cys_2_His_2_-Cys motifs as predicted (Iuchi et al., 2007). **(B)** Phylogenic analysis of SbSTOP1s and the above homologous proteins. The phylogenetic tree was constructed according to the neighbor-joining method using MEGA 5.1.

Using the amino acid sequences of AtSTOP1 and OsART1 as queries, four sweet sorghum *STOP1*-like genes, named *SbSTOP1a* (Sb01g001950.1), *SbSTOP1b* (Sb04g023670.1), *SbSTOP1c* (Sb07g023890.1), and *SbSTOP1d* (Sb03g041170.1), were identified in the sorghum genome database. The *SbSTOP1a, SbSTOP1b, SbSTOP1c*, and *SbSTOP1d* coding regions are 795, 1185, 1290, and 1560 bp, respectively, and they encode proteins of 264, 394, 429, and 519 amino acids, respectively. All SbSTOP1s contain four putative Cys_2_His_2_ zinc finger domains that resemble those of AtSTOP1, OsART1 and other homologs in different species (**Figure 1A**). SbSTOP1d shows the highest similarity to AtSTOP1 and OsART1, with identities of 54.9 and 48.5%, respectively, while SbSTOP1a, SbSTOP1b, and SbSTOP1c share relatively lower identities with AtSTOP1 and OsART1 (**Figure 1A**). Phylogenetic analysis revealed that SbSTOP1d clusters closely with AtSTOP1 and OsART1 compared to the other three SbSTOP1s. SbSTOP1b and SbSTOP1c cluster more closely with AtSTOP2 (**Figure 1B**).

### Detection of *SbSTOP1* Expression Patterns

**FIGURE 2:**
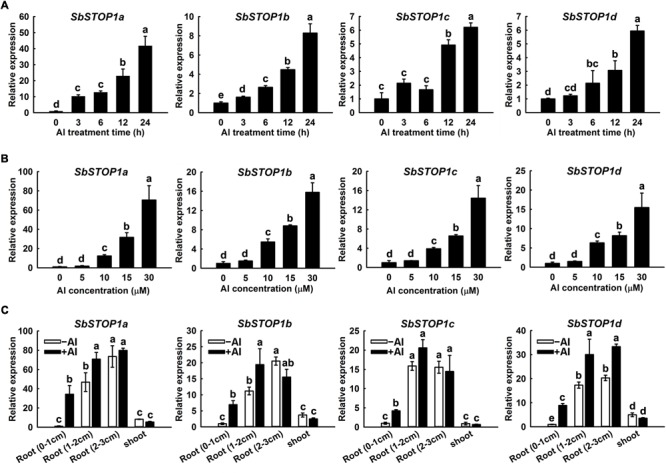
Quantitative real-time PCR analysis of *SbSTOP1s* expression profiles. **(A)** Relative expression of *SbSTOP1a, SbSTOP1b, SbSTOP1c*, and *SbSTOP1d* in sweet sorghum (*Sorghum bicolor*) root apices (0–1 cm) in response to 15 μM Al for different treatment times. **(B)** Relative expression of *SbSTOP1a, SbSTOP1b, SbSTOP1c*, and *SbSTOP1d* in root apices (0–1 cm) exposed to different Al concentrations for 24 h. **(C)** Relative expression of *SbSTOP1a, SbSTOP1b, SbSTOP1c*, and *SbSTOP1d* in root apices (0–1 cm), basal roots (1–2 cm), roots (2–3 cm) and shoots in the absence (–Al) or presence (+Al, 15 μM) of Al stress for 24 h. Data represent the means ± SD from three independent biological replicates. Columns with different letters are significantly different at *P* < 0.05.

The expression patterns of the SbSTOP1s were investigated using quantitative real-time PCR. A time-course experiment indicated that Al induced a gradual increase in *SbSTOP1a, SbSTOP1b, SbSTOP1c*, and *SbSTOP1d* expression in root apices (0–1 cm) during the entire 24 h Al treatment, though with different transcript abundances (**Figure 2A**). In addition, the four *SbSTOP1s* showed increased transcriptional abundances in a dosage-dependent manner when the roots were exposed to increasing external Al concentrations for 24 h (**Figure 2B**). All *SbSTOP1s* were mainly expressed in roots rather than shoots, and their expression levels in basal roots (1–2 cm) and roots (2–3 cm) were higher than that detected in root apices (0–1 cm) regardless of Al stress (**Figure 2C**). Al stress induced increasing *SbSTOP1s* expression in roots (especially in root apices), but there was no detectable effect on the expression of *SbSTOP1s* in shoots (**Figure 2C**). We also compared the expression of the four *SbSTOP1s* under Al stress with their expression under other metal and proton stress. The expression of *SbSTOP1c* was specifically induced by Al stress, while the expression of *SbSTOP1a, SbSTOP1b*, and *SbSTOP1d* was induced only by Al and Cd stress but not by other metals (Supplementary Figures 1A–D). In addition, as shown in Supplementary Figure 2, the expression of *SbSTOP1a, SbSTOP1b*, and *SbSTOP1c* was increased when the pH value of the treatment solution decreased. A notable exception was the expression of *SbSTOP1d*, which was relatively unaffected by low pH stress, similar to *OsART1* (Yamaji et al., 2009).

### The Subcellular Localization and Transcriptional Ability of SbSTOP1s

**FIGURE 3:**
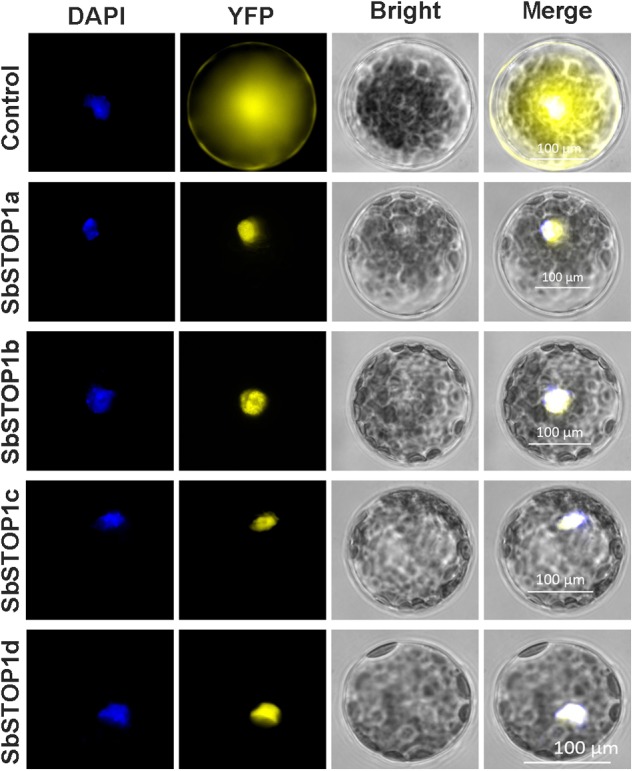
Subcellular localization of SbSTOP1s. Transient expression of YFP-SbSTOP1a, YFP-SbSTOP1b, YFP-SbSTOP1c, and YFP-SbSTOP1d fusion proteins and YFP control in *Arabidopsis* protoplast. DAPI, nuclear signal; YFP, YFP fluorescence; Bright, bright field. Scale bar indicates 100 μm.

The main transcriptional characteristics of the SbSTOP1s were examined, including the subcellular localization, transcriptional activity and DNA-binding property. *YFP-SbSTOP1a, YFP-SbSTOP1b, YFP-SbSTOP1c*, and *YFP-SbSTOP1d* fusion genes under the control of the cauliflower mosaic virus *35S* promoter were transiently introduced into *Arabidopsis* protoplasts. As shown in **Figure 3**, the YFP-SbSTOP1a, YFP-SbSTOP1b, YFP-SbSTOP1c, and YFP-SbSTOP1d fusion proteins were strictly localized to the nucleus, while the control YFP protein was distributed throughout the cytosol and nucleus.

**FIGURE 4:**
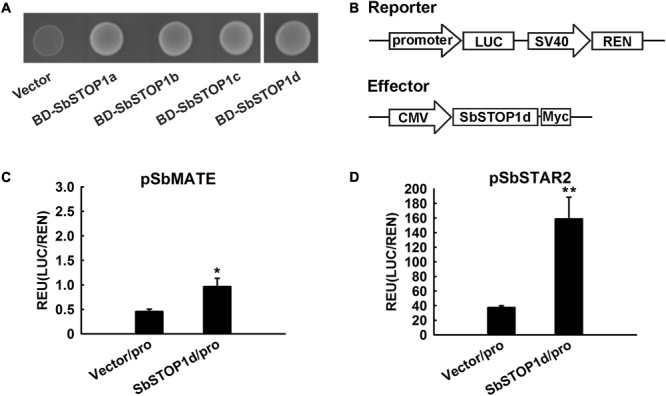
Transcriptional characteristic analysis of SbSTOP1s. **(A)** Transcriptional activity of SbSTOP1s in yeast. Y2HGold yeast strain carrying fused protein of GAL4 DNA-binding domain and SbSTOP1a (BD-SbSTOP1a), BD-SbSTOP1b, BD-SbSTOP1c, BD-SbSTOP1d or BD alone (vector) were cultured on SD-Trp-His medium. **(B)** Schematic diagram of the reporter and effector used in the HEK293 coexpression system. Promoter, *SbMATE* or *SbSTAR2* promoter (–2039 bp and –1963 bp, respectively); LUC, firefly luciferase reporter; REN, *Renilla* luciferase reporter as internal control; SV40 and cytomegalovirus (CMV), two promoters commonly used in mammalian expression vectors to drive gene expression; Myc, protein tag. **(C,D)** Transcriptional regulation of *SbMATE* **(C)** and *SbSTAR2* **(D)** by SbSTOP1d in HEK293 cells. Luciferase activity of reporter (LUC) driven by the promoters (pro) of *SbMATE* **(C)** and *SbSTAR2* **(D)** was normalized to the internal control reporter (REN). Data represent the means ± SD from three independent biological replicates. Asterisk (^∗^) represents significant differences from the vector-only control at *P* < 0.05. Asterisks (^∗∗^) represent significant differences from the vector-only control at *P* < 0.01.

The transcriptional activity of SbSTOP1s was assessed in the yeast expression system. SbSTOP1a, SbSTOP1b, SbSTOP1c, and SbSTOP1d were fused to the GAL4 DNA-BD. The resulting plasmids were transformed into the Y2HGold yeast strain with a His auxotrophic marker. As observed in **Figure 4A**, yeast cells carrying BD-SbSTOP1a, BD-SbSTOP1b, BD-SbSTOP1c, and BD-SbSTOP1d grew well in SD medium without His. In contrast, yeast cells containing the GAL4 DNA-BD alone did not. These results indicated that all four SbSTOP1s have transcriptional activity.

We further investigated the DNA-binding property of SbSTOP1d due to its high similarity to AtSTOP1 and OsART1 (**Figure 1**). *SbMATE* (*Sb03g043890*), the first and also one of the few reported Al tolerance genes in sorghum (Magalhaes et al., 2007), and *SbSTAR2* (*Sb09g001990*), an ortholog of *OsSTAR2* that is transcriptionally regulated by OsART1 (Yamaji et al., 2009; Tsutsui et al., 2011), were both examined as potential downstream genes using the HEK293 coexpression system (Gao et al., 2015) and a dual-luciferase reporter assay. We introduced the *SbMATE*/*SbSTAR2* promoter to drive the firefly luciferase reporter gene with the *Renilla* luciferase gene as an internal control (**Figure 4B**). As an effector, full-length SbSTOP1d under the control of the cytomegalovirus (CMV) promoter (**Figure 4B**) was co-transformed with the above reporter into HEK293 cells, and luciferase activity was detected. Both the *SbMATE* and *SbSTAR2* promoter-driven reporters showed higher luciferase activity in the presence of the SbSTOP1d effector compared to the vector-only effector, though the *SbSTAR2* promoter-driven reporter showed higher absolute value and significant differences at *P* < 0.01 compared with the *SbMATE* promoter-driven reporter (**Figures 4C,D**). SbSTOP1a, SbSTOP1b, and SbSTOP1c also showed weak or positive effects on the expression of *SbMATE* (Supplementary Figure 3). These results demonstrated that SbSTOP1d interacts with the *SbSTAR2* and *SbMATE* promoters to act as a transcriptional activator.

### SbSTOP1d Interacts With Itself or SbSTOP1b in Plants

**FIGURE 5:**
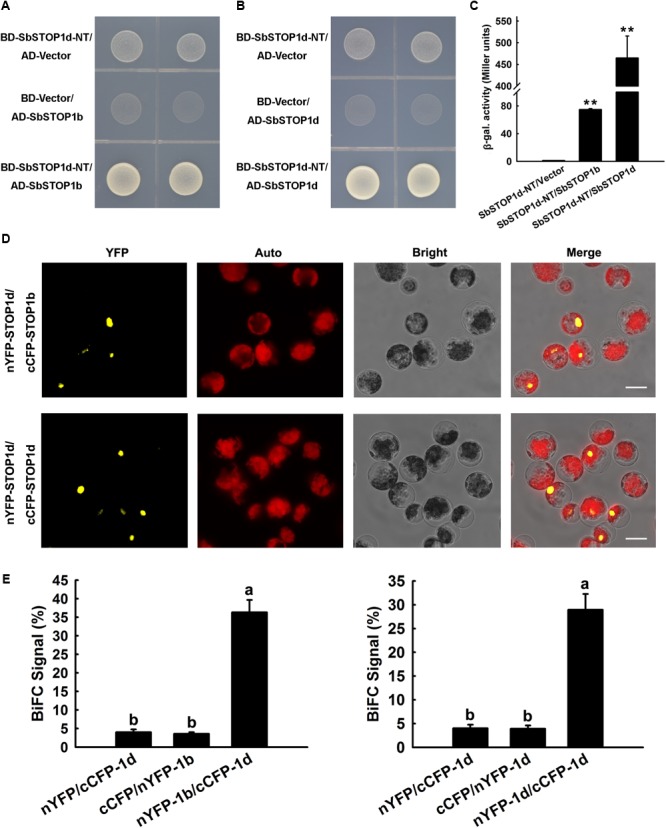
SbSTOP1d interacted with SbSTOP1b and SbSTOP1d itself. **(A,B)** Yeast Two-hybrid assays showing the interactions of BD-SbSTOP1d-NT with AD-SbSTOP1b **(A)** and BD-SbSTOP1d-NT with AD-SbSTOP1d **(B)**. **(C)** β-galactosidase assays showing the above protein interactions quantitatively. Data represent the means ± SD from three independent biological replicates. Asterisks (^∗∗^) represent significant differences in comparison to control at *P* < 0.01. **(D)** BiFC assays showing the association of SbSTOP1d and SbSTOP1b and the self-association of SbSTOP1d in *Arabidopsis* protoplasts. YFP, YFP fluorescence; Auto, autofluorescence; Bright, bright field. Scale bar indicates 100 μm. **(E)** The percentage of protoplasts that exhibit BiFC fluorescence signals was calculated. 1b, SbSTOP1b; 1d, SbSTOP1d. Data represent the means ± SD from three independent biological replicates. Columns with different letters are significantly different at *P* < 0.05.

We performed a yeast two-hybrid assay to screen for proteins that interact with SbSTOP1d, with the N-terminal fragment of SbSTOP1d [SbSTOP1d-NT, 1-275 aa, truncated before the zinc finger (ZF) domain] as bait, since its autoactivation could be readily suppressed by adding 3 mM 3-AT in SD medium (Supplementary Figure 4). Interestingly, the potential interaction proteins of SbSTOP1d included SbSTOP1d itself and SbSTOP1b. Yeast two-hybrid validation indicated that yeast cells co-transformed with SbSTOP1d-NT and SbSTOP1b grew well on SD/-Trp-Leu-His medium (**Figure 5A**). In addition, yeast cells containing SbSTOP1d-NT and SbSTOP1d showed similar result as the above (**Figure 5B**). Moreover, β-galactosidase assays showed that the β-galactosidase activities of yeast cells co-transformed with SbSTOP1d-NT and SbSTOP1b, and with SbSTOP1d-NT and SbSTOP1d were approximately 80 times and 450 times that of the control (**Figure 5C**). These results demonstrated that SbSTOP1d can interact with SbSTOP1d itself and SbSTOP1b at the N-terminal region (1–275 aa, not include ZF domain). SbSTOP1a and SbSTOP1c, nevertheless, showed no interaction with SbSTOP1d (Supplementary Figure 5). We further tested whether SbSTOP1d could interact with SbSTOP1b or itself in plant cells using the BiFC assay as described previously (Meng et al., 2013). SbSTOP1d was fused to the N-terminal fragment of YFP or to the C-terminal fragment of CFP, and SbSTOP1b was fused to the C-terminal fragment of CFP. Different pairs of constructs were co-transformed into *Arabidopsis* protoplasts while the protein–protein interaction was observed under a microscope (**Figure 5D**) and analyzed semi-quantitatively by measuring the percentage of cells that showed reconstituted YFP activity (**Figure 5E**). These results demonstrated that SbSTOP1d interacted with SbSTOP1d itself (self-association) as well as with SbSTOP1b in plants (**Figures 5D,E**), suggesting that SbSTOP1d might function as a homo- and/or heterodimer in plants. The homo- and/or heterodimerization of SbSTOP1d might facilitate its specificity and DNA-binding affinity, since this is a strategy used by other transcription factors (Crossley et al., 1995; Jakoby et al., 2002; Xu et al., 2015).

### *SbSTOP1d* Overexpression in *Arabidopsis* Confers Aluminum Tolerance

**FIGURE 6:**
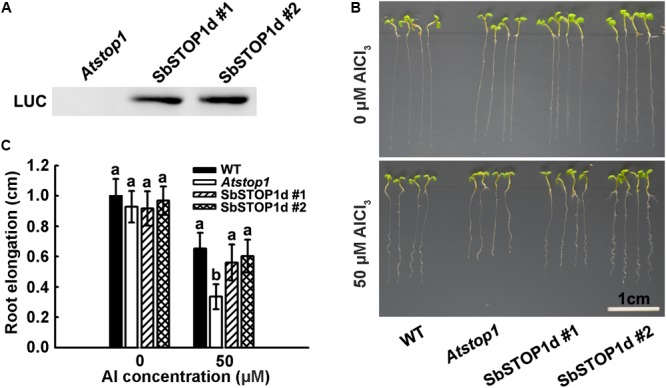
Transgenic *Arabidopsis* expressing *SbSTOP1d* shows improved tolerance to Al stress. **(A)** Immunoblot analysis of LUC-SbSTOP1d fusion protein in two independent complemented lines. **(B)** Al-sensitive phenotype of WT (Col4), *Atstop1* and *SbSTOP1d* complemented lines. Five-day-old seedlings were precultured on solid MS medium, then transferred to solid medium containing 4.3 mM CaCl_2_ and 3% sucrose at pH 4.5, with or without 50 μM AlCl_3_. **(C)** Root elongation of WT (Col4), *Atstop1* and *SbSTOP1d* complemented lines with or without Al treatment. Data are means of 20 replicates ± SD. Columns with different letters indicate significant differences between various plants under each Al treatment at *P* < 0.05.

The primary symptom of Al toxicity is a rapid inhibition of root growth (Foy, 1988; Kochian et al., 2004). The *Atstop1* mutant showed a root inhibition phenotype under Al stress, while the overexpression of *PpSTOP1, PnSTOP1*, or *NtSTOP1* in the *Atstop1* mutant could recover the Al- sensitive phenotype to varying extents (Ohyama et al., 2013; Fan et al., 2015). To further examine the function of SbSTOP1d, we introduced *LUC-SbSTOP1d* under the control of the CaMV 35S promoter in the *Atstop1* mutant background (SALK 114108). After a three-primer PCR-based genotyping (Supplementary Figure 6) and an immunoblot analysis for the LUC-SbSTOP1d fusion protein (**Figure 6A**), two independent complemented lines expressing SbSTOP1d were selected for phenotypic analysis. As shown in **Figures 6B,C**, the root growth of the WT, *Atstop1*, and two complemented lines was similar in the absence of Al. In the presence of Al, the root growth of WT was inhibited, with a relative root elongation (RRE) of 65%. *Atstop1*, which is sensitive to Al, had only 35% RRE, and in contrast, the two SbSTOP1d complemented lines greatly recovered the Al sensitivity characteristic of the *Atstop1* mutant, with 55 and 60% RRE, respectively. These results indicated that heterologous expression of *SbSTOP1d* improved the Al tolerance of the transgenic plants.

## Discussion

Aluminum can be a beneficial element for some plant species at low concentrations. At pH values below 5, aluminum concentration (Al^3+^) rises sharply, inhibiting root growth and function, which leads to significant reductions in crop yields (Foy, 1983; Liu et al., 2014; Bojórquez-Quintal et al., 2017; Moreno-Alvarado et al., 2017). Transcription factors, such as STOP1 and WRKY46 in *Arabidopsis*, ART1 and ASR5 in rice play important roles in Al signal perception and transduction (Sawaki et al., 2009; Yamaji et al., 2009; Ding et al., 2013; Arenhart et al., 2014; Xu et al., 2017). Moreno-Alvarado et al. (2017) recently reported for the first time the induction of transcription factor *NAC* gene expression in Al-treated rice plants. Among them, STOP1-like proteins have been shown to be key transcription factors and investigated in many plant species, including *Arabidopsis* (AtSTOP1, AtSTOP2), rice (OsART1), tobacco (NtSTOP1), eucalyptus (EguSTOP1), and rice bean (VuSTOP1) (Iuchi et al., 2007; Yamaji et al., 2009; Ohyama et al., 2013; Kobayashi et al., 2014; Sawaki et al., 2014; Fan et al., 2015), yet a STOP1-like protein has never been characterized in sorghum. We isolated four sweet sorghum genes, *SbSTOP1a, SbSTOP1b, SbSTOP1c*, and *SbSTOP1d*, encoding proteins containing four conserved C_2_H_2_ zinc finger domains, similar to other homologous proteins (**Figure 1**). Compared with the other three SbSTOP1s, SbSTOP1d shares higher similarity with AtSTOP1/OsART1 (**Figure 1**), its expression level was relatively higher under Al stress (data not shown) and it was specifically affected by Al stress but not low pH stress (Supplementary Figure 2D), thus, SbSTOP1d was further investigated and confirmed to be effective for Al tolerance in plants (**Figure 6**).

As previously reported, the expression levels of *AtSTOP1* and *OsART1* were not significantly affected by Al (Iuchi et al., 2007; Yamaji et al., 2009). However, in this study, the expression of *SbSTOP1a, SbSTOP1b, SbSTOP1c*, and *SbSTOP1d* in roots was significantly induced by Al in a time- and Al concentration-dependent manner (**Figure 2**). These results suggested that SbSTOP1s could respond to Al toxicity as early as transcriptional regulation and that different Al response mechanisms may exist between SbSTOP1s and AtSTOP1/OsART1. *VuSTOP1* shares similar expression patterns with the *SbSTOP1s*, since its expression is induced by Al stress, but VuSTOP1 expression is also affected by low pH (Fan et al., 2015). In sweet sorghum, *SbSTOP1a, SbSTOP1b*, and *SbSTOP1c* expression were induced by low pH (pH 3.5), but *SbSTOP1d* expression showed little change under proton stress (Supplementary Figure 2). Thus, it is possible that different STOP1-like proteins from various species or even from the same species differ in function. In addition, Al- and low pH- tolerance seem to be regulated by different gene groups that belong to the STOP1-regulated system (Kobayashi et al., 2014). Therefore, in this study, we focused on SbSTOP1d for further functional analysis due to its high sequence identity to AtSTOP1 and OsART1 and because its expression was specifically affected by Al toxicity but not proton stress. Further research could be conducted to investigate the detailed characterizations of different SbSTOP1s in sweet sorghum.

All SbSTOP1s localized to the nucleus (**Figure 3**), and this agreed with the expectation for transcription factors. The four SbSTOP1s displayed transcriptional activity. SbSTOP1d-CT (276–519 aa, including ZF domains) showed stronger transcriptional activity than that of SbSTOP1d-NT (1–275 aa), which could not be inhibited with 15 mM 3-AT in SD medium (Supplementary Figure 4).

STOP1-like proteins generally regulate the transcription of Al tolerance genes by binding to the *cis*-acting element of the promoter. OsART1 regulates multiple genes implicated in Al tolerance, and most of these genes (e.g., *OsSTAR1, OsSTAR2*) possess a *cis*-acting element as GGN(T/g/a/C)V(C/A/g)S(C/G) in their promoter (Yamaji et al., 2009; Tsutsui et al., 2011). AtSTOP1 also regulates several genes such as *AtALMT1* and *AtMATE1* in response to Al toxicity (Liu et al., 2009; Sawaki et al., 2009). Therefore, we tested whether SbSTOP1d regulates the transcription of two typical Al-associated genes, *SbMATE* (involved in Al exclusion mechanisms) and *SbSTAR2* (involved in Al tolerance mechanisms). Both genes contain the above putative *cis*-acting element in their promoters. SbSTOP1d showed a positive effect on the expression of *SbMATE* and *SbSTAR2* (**Figures 4C,D**), though the *SbMATE* promoter-driven reporter showed relatively lower luciferase activity than the *SbSTAR2* promoter-driven reporter did. Similarly, VuSTOP1 can bind only weakly to the promoter of *VuMATE* (Fan et al., 2015). It was reported that the promoter of *SbMATE* harbored a tourist like miniature inverted repeat transposable element (MITE). The copy number (sequence repeats) of this MITE, which varied in different sorghum accessions, was positively correlated with Al tolerance (Magalhaes et al., 2007). Thus, the expression level of *SbMATE* in the sweet sorghum cultivar we used may also be regulated by this transposable element. It is unlikely, but we cannot exclude the possibility that there are some other *cis*-acting elements away from the tested promoter (-2039 bp) of *SbMATE*, since VuSTOP1 can also interact with a DNA sequence lacking the putative GGN(T/g/a/C)V(C/A/g)S(C/G) *cis*-acting element (Fan et al., 2015). In addition, even though an increasing expression level of SbSTOP1s was induced by Al (**Figure 2**), some post-translational modifications may restrict the transcriptional activity of SbSTOP1. These modifications, such as protein phosphorylation, are frequently involved in the activation of transcription factors in response to biotic and abiotic stress, e.g., tomato *PSEUDOMONAS* TOMATO RESISTANCE (PTO) kinase phosphorylates PTI4 to increases the DNA-binding ability of PTI4 (Singh et al., 2002); Phosphorylation of ABA-responsive element binding proteins (AREB) was suggested to be involved in their activation (Uno et al., 2000). Thus, complex SbMATE regulation pathways may exist in sorghum. SbMATE-dependent citrate excretion is an important Al exclusion mechanism in sorghum (Magalhaes et al., 2007), while our study suggested that the SbSTOP1-dependent Al tolerance mechanism may blaze another trail, i.e., SbSTOP1 transcriptionally regulates *SbSTAR2* (**Figure 4D**) to fulfill its Al resistance function.

Homo- and/or heterodimerization of transcription factors occurs frequently to facilitate their function at diverse promoters or bring together/stabilize two regulatory elements. Plant basic-leucine zipper (bZIP) transcription factors form homodimers or heterodimers to bind DNA and *trans*-activate downstream gene expression (Schindler et al., 1992; Jakoby et al., 2002). Several types of zinc-finger motifs in transcription factors function as parts of DNA-binding and protein–protein interaction domains, e.g., GATA-1 in erythroid cells self-associates mediated by its zinc finger domain to influence transcription (Crossley et al., 1995). These studies provide a clue that the self-association of SbSTOP1d and association of SbSTOP1d and SbSTOP1b may also be beneficial for the DNA-binding property of SbSTOP1d. Differing from those of other zinc finger TFs, the association of SbSTOP1d itself (or with SbSTOP1b) in the yeast assays occurred at the N-terminal region, which lacks zinc finger domains (**Figures 5A–C**). In addition, SbSTOP1b shows the highest identity with AtSTOP2, except for SbSTOP1c in the sorghum genome database, and closely clusters with AtSTOP2 (**Figure 1B**). AtSTOP2, a homolog of AtSTOP1, was reported to activate transcription of some of the genes regulated by AtSTOP1 (Kobayashi et al., 2014), while the regulatory roles (individual roles or possible partnership) of AtSTOP1 and AtSTOP2 in Al- and low pH-tolerance have not yet been clarified. In the present study, SbSTOP1d and SbSTOP1b, as the homologous proteins of AtSTOP1 and AtSTOP2, respectively, displayed protein-protein interaction (**Figures 5A,C**). This result may help to further clarify the complex signal transduction pathways of STOP1-like proteins in response to Al and/or proton stress.

Complementation assays were conducted introducing multiple *AtSTOP1* orthologous genes in the *Atstop1* mutant background, and the results varied. Overexpression of *PpSTOP1* and *PnSTOP1* could almost fully or partially rescue the Al-sensitive phenotype of *Atstop1*, while CsSTOP1 was somewhat effective in transgenic lines (Ohyama et al., 2013; Sawaki et al., 2014). Here, SbSTOP1d greatly recovered the Al-sensitive phenotype of the *Atstop1* mutant, with nearly 90% of the root elongation of WT, demonstrating the Al tolerance function of SbSTOP1d in plants (**Figure 6**).

In summary, we have identified four STOP1-like genes (*SbSTOP1a, SbSTOP1b, SbSTOP1c*, and *SbSTOP1d*) in sweet sorghum that encode C_2_H_2_ zinc finger transcription factors. The expression of all four genes in roots was upregulated by Al stress. Heterologous expression of *SbSTOP1d* in *Atstop1* enhanced the Al tolerance of transgenic plants. SbSTOP1d interacted with itself (self-association) and SbSTOP1b in plants. These results provide a complete characterization of the SbSTOP1s in sweet sorghum and extend the understanding of STOP1-like transcription factors regulating Al tolerance in different plant species.

## Author Contributions

ZY designed the research and revised the manuscript. SH, JG, YL, KG, SY, and MZ conducted the experiments. SH and JY analyzed the data. JG wrote the manuscript.

## Conflict of Interest Statement

The authors declare that the research was conducted in the absence of any commercial or financial relationships that could be construed as a potential conflict of interest.

## Footnotes

1 http://pgsb.helmholtz-muenchen.de/plant/sorghum/
