# Supplementary material for: Identification of STOP1-Like Proteins Associated With Aluminum Tolerance in Sweet Sorghum (Sorghum bicolor L.)
Source: Front Plant Sci. 2018 Feb 28;9:258. doi: 10.3389/fpls.2018.00258 (PMC5835670; doi:10.3389/fpls.2018.00258)
Supplement: Supplementary file 1 [file Data_Sheet_1.docx]

Supplementary Material

Identification of STOP1-like Proteins Associated with Aluminum Tolerance in Sweet Sorghum (*Sorghum bicolor* L.)

Sheng Huang^†^, Jie Gao^†^, Jiangfeng You, Yanan Liang, Kexing Guan, Siqi Yan, Meiqi Zhan, Zhenming Yang^*^

*** Correspondence:** Zhenming Yang: zmyang@jlu.edu.cn

^†^These authors have contributed equally to this work.

## Supplementary Table

**Supplementary Table 1.** Sequences of primers for quantitative real-time PCR.

| **Gene** | **Primer** | **Sequence (5′–3′)** |
| --- | --- | --- |
| *SbSTOP1a* | SbSTOP1a-F | ACGCTGTTACCAATGCTGTCG |
|  | SbSTOP1a-R | CTCACTGCCTCGGTGTCCAT |
| *SbSTOP1b* | SbSTOP1b-F | ATCACTAAGCCAAACAAG |
|  | SbSTOP1b-R | ATTGCCTTCTTCAAATCC |
| *SbSTOP1c* | SbSTOP1c-F | CGCCAAGAACCACTACAAG |
|  | SbSTOP1c-R | GAGAAGTGCTTGCGGTTG |
| *SbSTOP1d* | SbSTOP1d-F | TTCGTCAATGGCAAGCA |
|  | SbSTOP1d-R | AACTGGAGGAGGGAGGC |
| *β-actin* | β-actin-F | CGACCTTACCGACTACCTCATG |
|  | β-actin-R | TCTTGGCAGTCTCCATCTCCT |

## Supplementary Figures


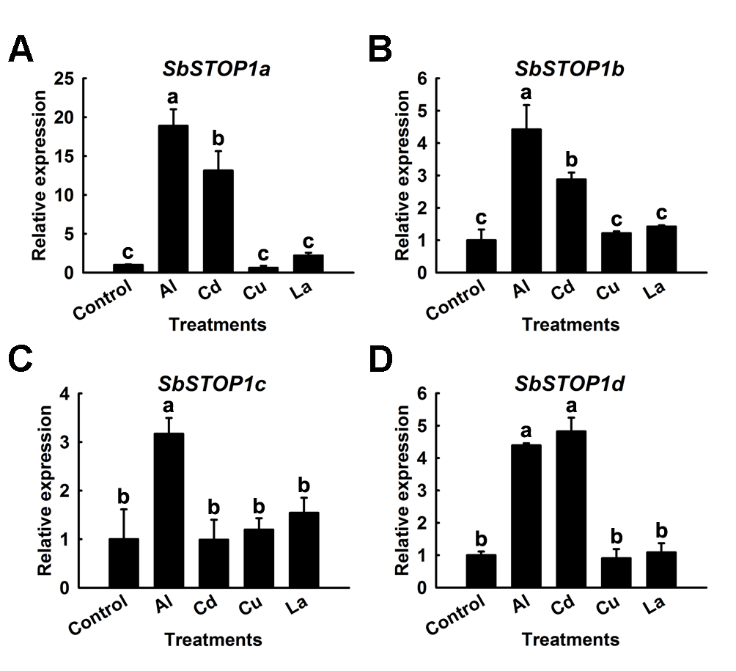


**Supplementary Figure 1.** Quantitative real-time PCR analysis of *SbSTOP1a*, *SbSTOP1b*, *SbSTOP1c* and *SbSTOP1d* expression in sweet sorghum (*Sorghum bicolor*) root apices in response to AlCl_3_ (15 μM), CdCl_2_ (10 μM), CuCl_2_ (0.5 μM) and LaCl_3_ (10 μM) for 24 h. Data represent the means ± SD from three independent biological replicates. Columns with different letters are significantly different at *P* < 0.05.


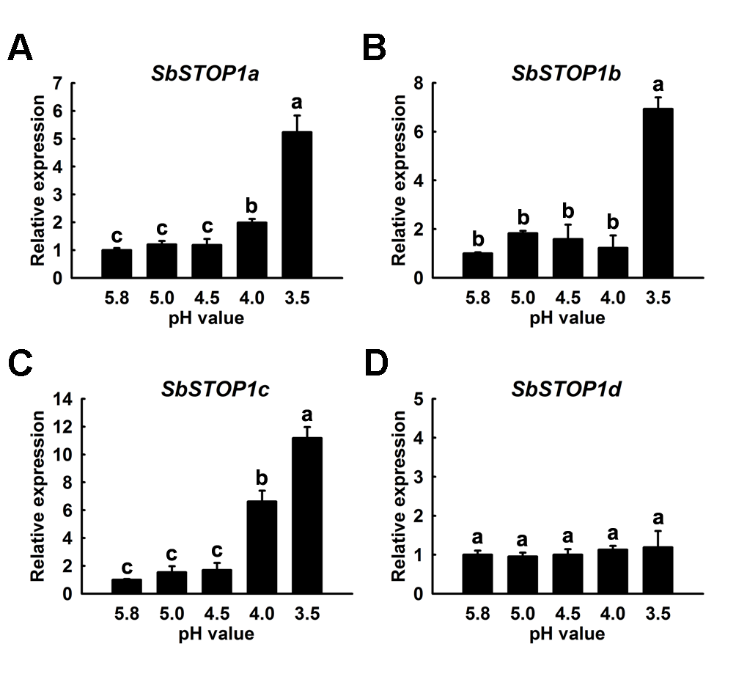


**Supplementary Figure 2.** Quantitative real-time PCR analysis of *SbSTOP1a*, *SbSTOP1b*, *SbSTOP1c* and *SbSTOP1d* expression in sweet sorghum (*Sorghum bicolor*) root apices in different pH conditions. Data represent the means ± SD from three independent biological replicates. Columns with different letters are significantly different at *P* < 0.05.

**
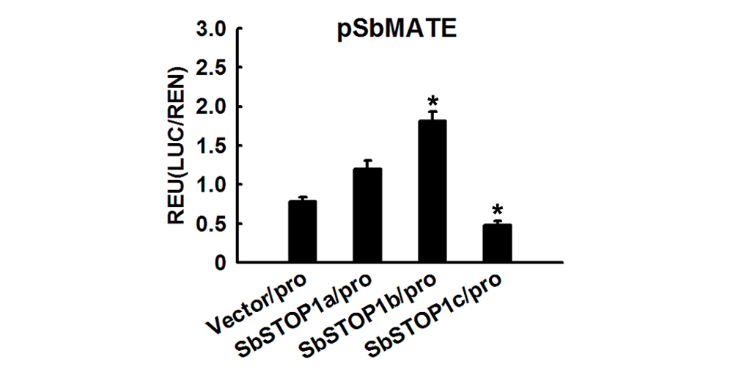
**

**Supplementary Figure 3.** Transcriptional regulation of *SbMATE*. SbSTOP1a, SbSTOP1b and SbSTOP1c showing different effect on the transcriptional regulation of *SbMATE* in HEK293 cells. Data represent the means ± SD from three independent biological replicates. Asterisk (*) represents significant differences from the vector-only control at *P* < 0.05.


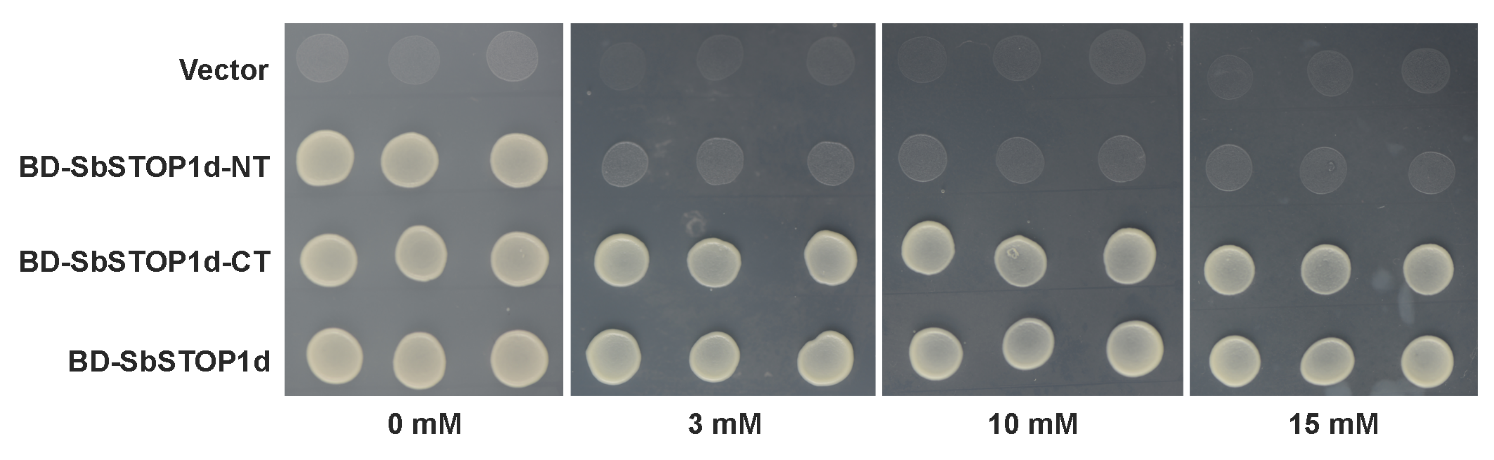


**Supplementary Figure 4.** Autoactivation detection of SbSTOP1d. N-terminal fragment (1-275 aa), C-terminal fragment (276-519 aa) and full length of SbSTOP1d were fused to GAL4 DNA-binding domain respectively, named BD-SbSTOP1d-NT, BD-SbSTOP1d-CT and BD-SbSTOP1d. The above constructs and BD only control (vector) were cultured on SD-Trp-His medium with 0 mM, 3 mM, 10 mM and 15 mM 3-AT, respectively.


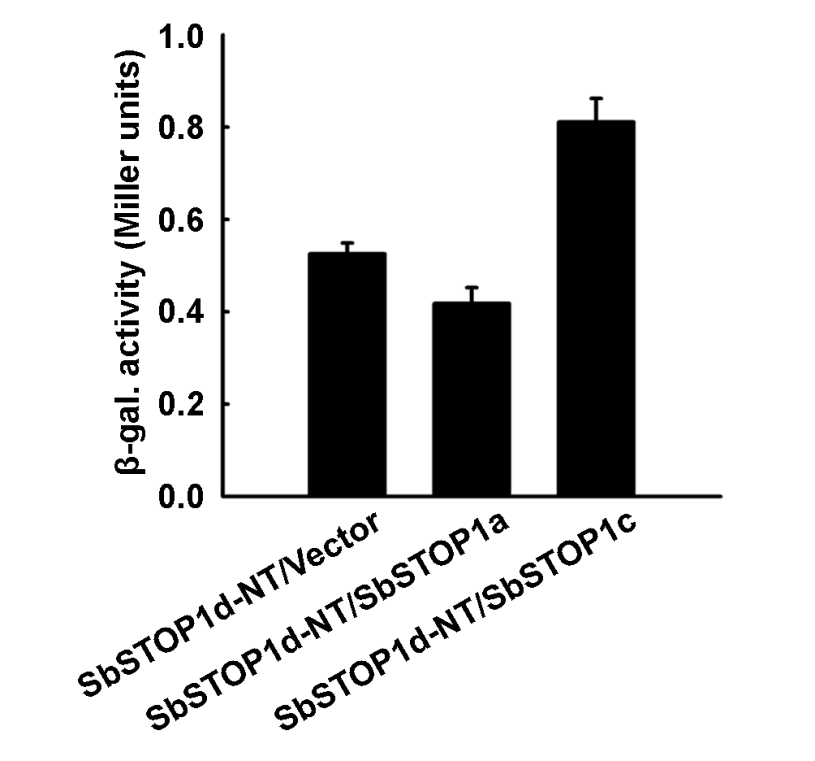


**Supplementary Figure 5.** SbSTOP1a and SbSTOP1c showed no interaction with SbSTOP1d in yeast cells. Data represent the means ± SD from three independent biological replicates.


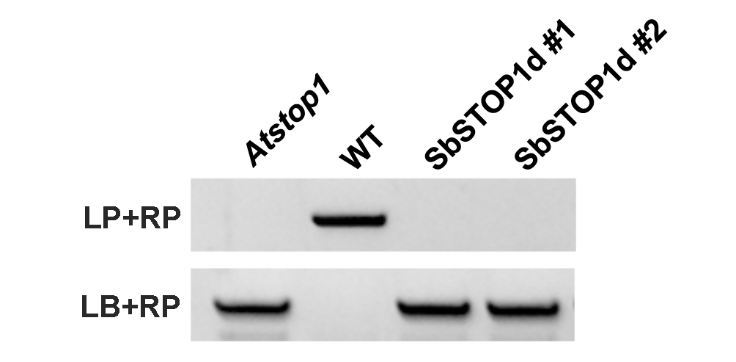


**Supplementary Figure 6.** A three-primer PCR-based genotyping of SbSTOP1d complemented lines. LP and RP, gene specific primers; LB, T-DNA insertion fragment primer.
